# Vaccination with a *Trypanosoma cruzi* Protein Extract Plus BCG∆BCG1419c Promotes a Balanced Th1/Th2 Immune Profile That Improves Control of Acute Chagas Disease in BALB/c Mice

**DOI:** 10.3390/microorganisms13112447

**Published:** 2025-10-25

**Authors:** Olivia Rodríguez-Morales, Minerva Arce-Fonseca, Dulce Mata-Espinosa, Alberto Aranda-Fraustro, José Luis Rosales-Encina, Mario Alberto Flores-Valdez

**Affiliations:** 1 Laboratorio de Inmunología Molecular y Protéomica, Departamento de Biología Molecular, Instituto Nacional de Cardiología Ignacio Chávez, Mexico City 14080, Mexico; 2 Laboratorio de Patología Experimental, Instituto Nacional de Ciencias Médicas y Nutrición Salvador Zubirán, Mexico City 14080, Mexico; 3 Departamento de Patología, Instituto Nacional de Cardiología Ignacio Chávez, Mexico City 14080, Mexico; arandafraustro@yahoo.com.mx; 4 Laboratorio de Biología Molecular, Departamento de Infectómica y Patogénesis Molecular, Centro de Investigación y de Estudios Avanzados del Instituto Politécnico Nacional, Mexico City 07360, Mexico; rosales@cinvestav.mx; 5 Biotecnología Médica y Farmacéutica, Centro de Investigación y Asistencia en Tecnología y Diseño del Estado de Jalisco, A. C., Guadalajara 44270, Mexico

**Keywords:** Chagas disease, *Trypanosoma cruzi*, BCG, adjuvant, vaccine, *Mycobacterium bovis*

## Abstract

Adjuvants in vaccine candidates against Chagas disease (ChD) have been tested with the aim of improving efficacy against this disease. *Trypanosoma cruzi* total protein extract (TcTPE) combined with *Mycobacterium bovis* BCG Pasteur strain ATCC 35734 or its isogenic derivative mutant BCGΔBCG1419c—in which the *BCG1419c* gene was deleted—were used as a vaccine formulation in BALB/c mice. After immunization and *T. cruzi* challenge, parasitological and clinical parameters of acute stage were recorded. Antibody titers, cytokine concentrations, macroscopic abnormalities, and histological analysis of experimental ChD were determined. The vaccine formulation with the combination of TcTPE and BCGΔBCG1419c, used as the adjuvant, reduced parasitemia by 50%, promoted a survival rate of 80%, improved the clinical status, favored greater body weight gain, induced high titers of specific anti-*T. cruzi* IgG antibodies and some cytokines, leading to a balanced Th1/Th2 immunological profile as well as a lower degree of inflammation and tissue damage (50% reduction). A good protective effect in the acute stage of experimental ChD was observed by favorably modulating the immune response and reducing heart and muscle damage, therefore, highlighting that the combination tested here for the control of ChD provides a promising basis that warrants further investigation to validate a future potential translation to humans.

## 1. Introduction

The Chagas disease (ChD), of which its causal agent is *Trypanosoma cruzi*, is transmitted by a hematophagous insect vector and is considered one of the most serious parasitic infections in Latin America (an endemic area of the disease) along with 21 other diseases that are part of the Neglected Tropical Diseases group according to the World Health Organization (WHO) [1]. Due to the constant migratory flow, in the last two decades, ChD has spread to other parts of the world such as the United States, Canada, Europe, Oceania, some African countries, the Eastern Mediterranean, and the Western Pacific [2]. Around 6 to 7 million people are infected worldwide, 75 million people are at risk of becoming infected, and there is an annual incidence of 56,000 cases with 12,000 deaths occurring annually due to ChD. According to data from the Centers for Disease Control and Prevention (CDC), there are around 300,000 people infected with *T. cruzi* in the United States and 80,000 infected in Europe [3,4].

Over the years, the use of adjuvants to increase the effectiveness of vaccine candidates against ChD has been reported with varied results, with some promising an adequate protective state [5,6,7]. Immunostimulant and adjuvant roles provided by *M. bovis* BCG have been reported to induce protection against *T. cruzi* infection and the severe presentation of chronic ChD; there was a decrease in the antibody levels and cardiac involvement in patients with chronic ChD who had been vaccinated with BCG in their childhood [8]; also, mice that received a BCG vaccination had better responses against severe forms of experimental ChD [9].

On the other hand, in the BCG∆BCG1419c strain, the *BCG1419c* gene-encoding cyclic diguanylate (c-di-cGMP) phosphodiesterase was deleted. C-di-GMP is a second messenger that is synthesized from guanosine triphosphate by the enzyme diguanylate cyclase and hydrolyzed by c-di-GMP phosphodiesterase. Removing the *BCG1419c* gene gives BCG Pasteur a greater capacity for in vitro biofilm production, tolerance to stress induced by reactive nitrogen species and to the action of antibiotics, increases autophagy in murine macrophages, increases T cell memory responses, and improves the control of tuberculosis pathology in animal models since it delays the progression of lung necrosis [10,11], possibly linked to changes in the expression of several antigenic proteins [12]. The differential immunological characteristics, as well as the greater persistence in tissues with respect to the wild strain, make this mutant a possible candidate as a promising vaccine adjuvant/platform against ChD.

In this context, the aim of this study was to compare the protective effects of two vaccine formulations against *T. cruzi* infection using a total protein extract from the parasite combined with the administration of *M. bovis* BCG Pasteur ATCC 35734 (wtBCG) or its derived mutant, BCG∆BCG1419c, with the deletion of the c-di-GMP phosphodiesterase-encoding gene, *BCG1419c*, as adjuvants.

## 2. Materials and Methods

### 2.1. Experimental Animals

Female BALB/c mice were bred and originated from the Laboratory Animal Production and Experimentation Unit of the Center for Research and Advanced Studies of the National Polytechnic Institute, Mexico City, Mexico. Groups of five 6-to 8-week-old, 20.8 ± 0.5 g on average, BALB/c mice were classified as follows and as shown in Table 1: (I) not-vaccinated, not-infected mice; (II) not-vaccinated, *T. cruzi*-infected mice; (III) total protein extract from *Trypanosoma cruzi* (TcTPE)-vaccinated mice and using the wild-type *M. bovis* BCG Pasteur ATCC 35734 strain as an adjuvant, not infected; (IV) TcTPE-vaccinated mice plus the wild-type *M. bovis* BCG Pasteur ATCC 35734 strain as an adjuvant, and *T. cruzi*-infected; (V) TcTPE-vaccinated mice and using the *M. bovis* BCG∆BCG1419c mutant strain as an adjuvant, not infected; (VI) TcTPE-vaccinated mice plus the *M. bovis* BCG∆BCG1419c mutant strain as an adjuvant, and *T. cruzi*-infected; (VII) TcTPE-vaccinated mice without any adjuvant, not infected; (VIII) TcTPE-vaccinated mice without any adjuvant, and *T. cruzi*-infected. Experiments were carried out in duplicate to confirm the reproducibility of our findings. Likewise, we inoculated female mice with the Ninoa *T. cruzi* strain (MHOM/MX/1994/Ninoa) to ensure a progressive, non-lethal infection, since males are more susceptible to ChD [13,14], as well as to be able to compare our current results with those obtained in previous studies [9]. The animal experimental protocol was carried out in accordance with the provisions of the Guide for Care and Use of Experimental Animals, National Institutes of Health [15] and the Mexican Official Standard (Norma Oficial Mexicana NOM-062-ZOO-1999. Technical specifications for the production, care and use of laboratory animals) [16].

### 2.2. Vaccine Inoculum and Adjuvants Preparation

A 30 mL culture of INC-9 isolate epimastigotes in LIT (Liver Infusion Tryptose) medium supplemented with 10% fetal bovine serum and 0.025% hemin with 10 days of incubation at room temperature was centrifuged at 12,000 rpm for 30 min at 4 °C (Thermo Fisher Scientific^®^ brand centrifuge, model SORVALL RC 6 Plus, and a Thermo Fisher Scientific^®^ brand aluminum rotor, model SLA-1500, Waltham, MA, USA). The pellet was resuspended with 30 mL of phosphate buffer (PBS 0.01 M, pH = 7.2) and centrifuged at 8000 rpm for 30 min; this wash was performed twice. Pellet was resuspended in 2 mL of 0.01 M PBS, and the parasites were submitted to sonication cycles (Sonics^®^ brand sonicator, model VCX 130 PB, with the CV18 probe, Newtown, CT, USA) at 100 mA every 2 min, 6 to 7 times, keeping the parasites on ice throughout the process. The cell lysate obtained was placed in 1.5 mL microcentrifuge tubes (Eppendorf^®^, Hamburg, Germany) and centrifuged (Sorvall™ DuPont^®^, model RMC-14, Wilmington, NC, USA) at 10,000 rpm for 30 min, and the supernatant containing the soluble *T. cruzi* proteins was recovered. Protein concentration was determined by the Lowry method using the UV/Visible spectrophotometer (Thermo Fisher Scientific^®^, model BioMate 3, Waltham, MA, USA). Aliquots of 10–15 µg of TcTPE were made in 100 µL sterile 0.01 M PBS in 0.6 mL microtubes and stored at 4 °C until use.

*M. bovis* BCG Pasteur ATCC 35734 and its derived mutant BCG∆BCG1419c strain were obtained under appropriate conditions as previously described [9]. The vaccine dose was confirmed by counting colony-forming units (CFU) on Middlebrook 7H10 agar plates after 3 weeks of incubation at 37 °C.

### 2.3. Mice Immunization

The vaccines were prepared at the time of administration by mixing 100 µL containing 10–15 µg of the TcTPE plus 10^4^ CFU of wtBCG or BCG∆BCG1419c strain in 100 μL each; a booster was given 21 days later in which 100 µL of each component were used in doses slightly lower than the first application, but maintaining the same ranges, as follows: 15 µg of the TcTPE, 4.8 × 10^4^ CFU of wtBCG or 4.1 × 10^4^ CFU of BCG∆BCG1419c for the first experiment and the first immunization; 10 µg of the TcTPE, 1.1 × 10^4^ CFU of wtBCG or 3.1 × 10^4^ CFU of BCG∆BCG1419c for the first experiment and the second immunization. In replicate trials, 15 µg of the TcTPE, 6.3 × 10^4^ CFU of wtBCG or 6.6 × 10^4^ CFU of BCG∆BCG1419c for the first immunization, and 10 µg of the TcTPE, 2 × 10^4^ CFU of wtBCG or 2.7 × 10^4^ CFU of BCG∆BCG1419c for the second immunization were used. These dosages were in accordance with previous studies [10,13,17,18] and based on the fact that booster administration is not recommended for BCG vaccination, as it increases the likelihood of more intense local reactions that are not considered severe. Furthermore, even though experimentally confirmed doses, which were prepared from different vaccine batches, explaining the variation in actual dose applied, were slightly different between BCG strains and replicate experiments, it was recently demonstrated that the efficacy of protection of BALB/c mice against *M. tuberculosis* was conserved when vaccine doses ranged between 3 × 10^3^ and 3 × 10^5^ CFU, which was the case for the doses employed in our work [19]. Vaccination was performed subcutaneously in the dorsal area at the level of the scapular region of each mouse using 0.5 mL insulin syringes, with an integrated 31 G × 8 mm needle and dead space of less than 0.005 mL.

### 2.4. Parasite Challenge and Serum Samples

On the 21st day after booster, mice were intraperitoneally injected with 250 blood trypomastigotes of the Ninoa *T. cruzi* strain (MHOM/MX/1994/Ninoa) resuspended in 200 μL of sterile saline solution using a 27 G × 13 mm insulin needle.

**Figure 1:**
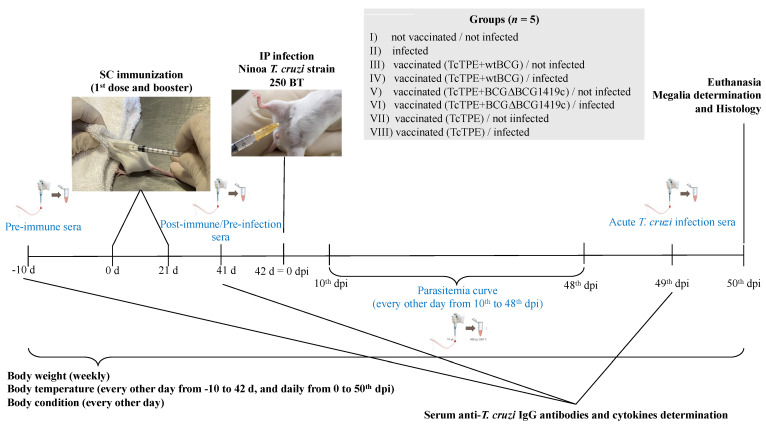
Timeline and interventions of the study. Eight groups of BALB/c mice were subcutaneously immunized twice at 21-day interval with 100 µL containing 10–15 µg of the TcTPE plus 10^4^ CFU of wtBCG or BCG∆BCG1419c strain in 100 μL each to evaluate the effectiveness of the two BCG strains as adjuvants. All procedures were performed in duplicate, and data showed in the subsequent figures are representative of the two independent experiments. SC = subcutaneous, IP = intraperitoneal, BT = blood trypomastigotes, d = days, dpi = day post-infection, TcTPE = total protein extract from *Trypanosoma cruzi*.

Blood samples were obtained from all experimental groups at three significant times during the experiment as shown in the timeline of the study (Figure 1): (i) prior to the start of the experiment (pre-immune sera), (ii) after the second vaccination (post-immune/pre-infection sera), and (iii) during the acute phase of ChD before euthanasia (acute *T. cruzi* infection sera), prior to the sacrifice and blood collection at the time of necropsy. The samples from the second time (post-immune/pre-infection sera) were obtained three hours after the subcutaneous (SC) application of the booster vaccine in order to obtain data for a better quantification of serum cytokine levels with an antigenic re-stimulation according to other murine model studies on vaccines against ChD [17,18].

Blood was collected in 1.5 mL microtubes without anticoagulant; these samples were kept at room temperature for 30 min (until clot retraction), centrifuged (Sorvall™ DuPont^®^, model RMC-14, Wilmington, NC, USA) at 3500 rpm for 15 min at 4 °C, and aliquots of 50 µL were then made into 0.6 mL microtubes, which were stored frozen at –20 °C until use.

### 2.5. Parasitemia and Survival Assays

Blood samples were collected to assess the parasitemia by the modified Petana method [20] and to construct a curve recording every other day starting at the 10th day post-infection (dpi) and concluding after registering three consecutive days of zero parasite counts; briefly, a 1:50 dilution was made by adding 10 μL of peripheral blood extracted through the caudal vein to 490 μL of saline solution (0.9% NaCl), and parasitemia was quantified in a Neubauer counting chamber by light microscopy.

Mice survival was monitored daily until the day of euthanasia during the acute phase (50th dpi). Animals that died outside of working hours underwent directed necropsy as soon as possible and the results were considered for further analyses. Body remains were disposed in accordance with the provisions of the Mexican Official Standard (Norma Oficial Mexicana NOM-087-ECOL-SSA1-2002: Environmental protection, Environmental health, Biological-infectious hazardous waste, and Classification and management specifications) [21].

### 2.6. Animal Clinical Examination

Three parameters were assessed: body weight, temperature, and physical condition. Body weight was recorded weekly using a scale (Sartorius^®^, model BL 1500 S, Göttingen, Lower Saxony, Germany). Body temperature was recorded every other day with a digital thermometer (Citizen^®^, model CTA303, Tokyo, Japan) in the ear flap of the mouse upon arrival of the animals, after the first and second vaccination, and daily after infection. Body condition was assessed every other day throughout the experiment by observing physical appearance using a scoring scale in which a numerical value was assigned for each clinical sign in the mouse as described previously [9]. Scores of 4 and 5 marked the humane endpoint in the animals; when the mice presented these indicators, euthanasia was applied according to the guidelines established by the NOM-062-ZOO-1999 [14].

### 2.7. Anti-T. cruzi Humoral Immune Response Evaluation

The humoral immune response was evaluated by detecting anti-*T. cruzi* IgG antibodies, as well as IgG1 and IgG2a subclasses using the ELISA technique, according to the protocol described previously [9]. As an internal control of the experiment, we verified whether *M. bovis* BCG served as an adjuvant by assessing the presence of IgG antibodies against a purified protein derivative (PPD) (PRONABIVE, *M. bovis* AN5, Mexico City, Mexico) of *M. bovis* as reported previously [9].

### 2.8. Serum Cytokines Determination

The flow cytometry technique was performed to identify the cytokines interferon (IFN) IFN-γ, interleukin (IL) IL-1β, IL-2, IL-4, IL-6, IL-10, IL-12, and IL-18, and the tumor necrosis factor (TNF) TNF-α at the time of euthanasia; the BioLegend^®^ LEGENDplex™ Mouse Inflammation Panel commercial kit (San Diego, CA, USA) was used following the manufacturer’s recommendations.

### 2.9. Macroscopic and Microscopic Findings

Euthanasia was performed in a CO_2_ chamber and directed necropsy was carried out to harvest organs targeted by ChD including the heart, skeletal muscle, esophagus, intestines, brain, lymph nodes and spleen. The lungs were also collected to analyze the possible effect of BCG strains on them. A description of the macroscopic findings was compiled, and the determination of megalia by calculating the organic indices in the acute stage of the infection was performed as described previously [9]. The organs were rinsed in sterile saline solution (0.9% NaCl), preserved in 10% formalin for at least 48 h and processed. In brief, the fixed organs were washed with deionized water, dehydrated, embedded in paraffin for histological sections with a 0.5-micron-thick microtome, stained with hematoxylin–eosin and observed under an optical microscope. For histopathological analysis, a scoring scale was used to evaluate the degree of tissue damage, with 1 point being the absence of alterations, and as the score progresses, there is a worsening of the histological structures, reaching grade 5 (Table 2).

### 2.10. Statistical Analysis

The data collected were analyzed using the GraphPrism 9.0 program, (GraphPad 9.5.1 version Software, San Diego, CA, USA). Means from parasitemia were compared by one-way ANOVA statistical test followed by the Dunnett’s post hoc test; survival was analyzed using the Kaplan–Meier test; physical condition, megalia, and histopathology data were compared by the nonparametric Kruskal–Wallis test with Dunn post hoc test; the weight, temperature, antibodies, and cytokines data were analyzed with one-way ANOVA through the Tukey post hoc test, and the values were considered significantly different when *p* ≤ 0.05.

## 3. Results

### 3.1. Beneficial Effect on Parasitemia and Survival upon Vaccination Using BCGΔBCG1419c as an Adjuvant

Blood-circulating parasite forms were detectable from 16th dpi in all groups, reaching maximum levels in all experimental groups between 28th and 30th dpi. The average parasite load for each group is shown in Table 3.

**Figure 2:**
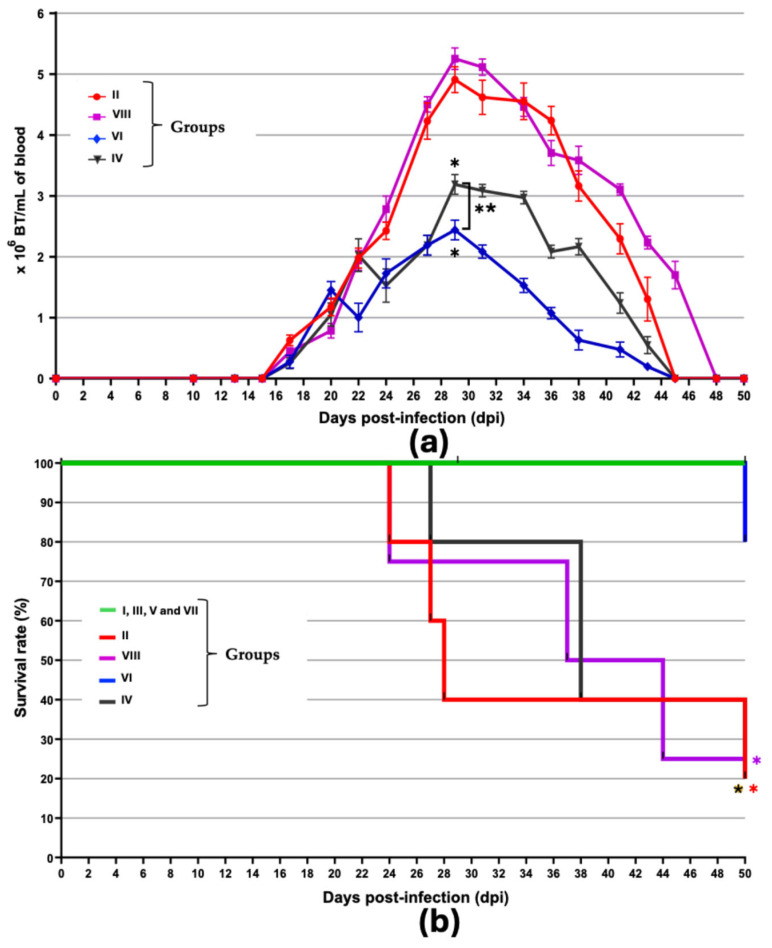
Parasitemia curve (**a**) and survival rate (**b**) of *T. cruzi*-infected mice, previously vaccinated. Values represented in (**a**) are the mean and standard deviation (S.D.) of the amount of blood trypomastigotes (BT) quantified in each mouse. Parasitemia peaks of groups IV, VI, and VIII were compared with group II (not vaccinated/infected mice) using the one-way ANOVA statistical test, followed by the Tukey post hoc test, and the differences were considered significant (*) when *p* ≤ 0.05. In addition, when comparing groups IV and VI with each other, a significant difference (**) was also found (*p* ≤ 0.05). In (**b**), the survival of groups II, IV, VI, and VIII was compared with respect to groups I, II, V, and VII (not infected mice); the Kaplan–Meier statistical test was used, followed by the Log-rank test (Mantel–Cox) to determine the existence of a significant difference (*) (*p* ≤ 0.05). Both assays are representative of duplicates performed independently. Group I = not vaccinated/not infected; group II = not vaccinated/infected; group III = TcTPE-vaccinated + wtBCG as adjuvant/not infected; group IV = TcTPE-vaccinated + wtBCG as adjuvant/infected; group V = TcTPE-vaccinated + BCG∆BCG1419c as adjuvant/not infected; group VI = TcTPE-vaccinated + BCG∆BCG1419c as adjuvant/infected; group VII = TcTPE-vaccinated/not infected; group VIII = TcTPE-vaccinated/infected.

The group of BALB/c mice vaccinated with TcTPE using BCGΔBCG1419c as an adjuvant (group VI) showed 50.3% reduction in parasitemia compared to group II (not vaccinated/infected), whereas the decrease was only 35% for group IV (TcTPE + wtBCG-vaccinated/infected), suggesting that immunization using *M. bovis* BCGΔBCG1419c as an adjuvant conferred greater protection by reducing parasitemia to a greater extent. It is important to note that group IV had a higher peak of parasitemia and that most animals (75%) died during the acute stage (Figure 2a).

Group VI was observed to have an 80% (4/5) survival rate, very similar to that of the healthy mice (group I) as well as the other three groups that only had immunizations with each of the vaccine formulations without receiving the *T. cruzi* challenge (groups III, V, and VII); in all these not-infected groups, survival rate was 100%, which confirmed the safety of the three vaccine formulations. In contrast, group VIII had a survival rate of only 25% (1/4), and groups IV and II showed 20% survival (1/5). These data demonstrate that the vaccine with TcTPE using BCGΔBCG1419c as an adjuvant (group VI) protected mice against mortality from acute ChD until euthanasia of the mice (Figure 2b). The median survival time (MST) was 28 days for group II, 38 days for the group in which the wtBCG strain was used as an adjuvant (group IV), and 40.5 days for the group vaccinated only with TcTPE without adjuvant (group VIII). For the rest of the groups, their MST was indefinite, which confirms the protection conferred by the BCGΔBCG1419c mutant strain when used as an adjuvant in vaccination against *T. cruzi*. In group VIII (TcTPE-vaccinated/infected), euthanasia was performed for humane reasons in two animals on the 37th and 44th dpi, respectively.

### 3.2. Vaccination Using BCGΔBCG1419c as an Adjuvant Caused Less Weight Loss than wtBCG and Prevented Fever and Physical Deterioration

**Figure 3:**
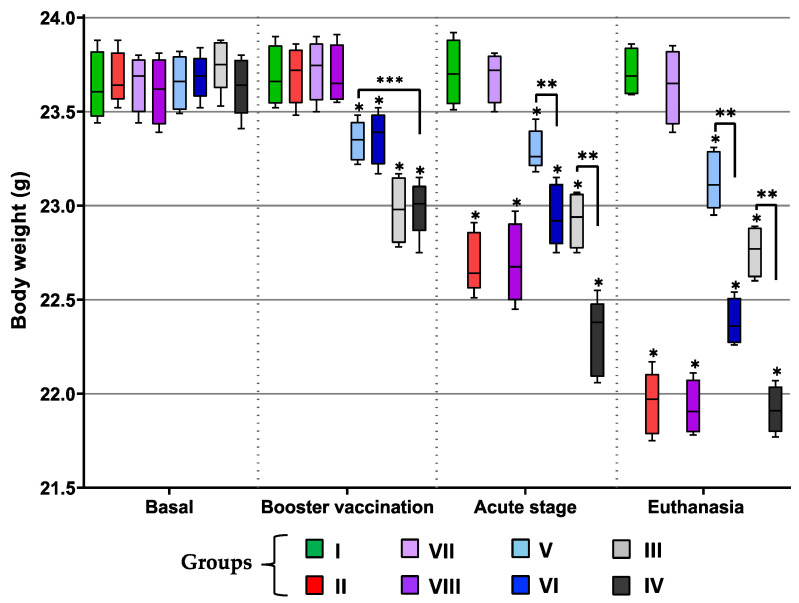
Body weight of BCG-immunized or not immunized mice and infected or not with *T. cruzi*. Values show the mean with S.D. for each group and are representative of two independent experiments with equivalent results. The values were grouped into four stages (see timeline for further reference, Figure 1): baseline, from −10 d to 0 d; booster vaccination, from 1 d to 42 d; acute stage, from 2nd to 48th dpi and euthanasia, from 48th to 50th dpi. Values from each time were analyzed through multiple comparisons among groups by two-way ANOVA test followed by Dunnett’s post hoc test, and significant difference is shown (*) when *p* ≤ 0.05 compared to the group I (healthy mice), (**) when comparing the groups immunized with wtBCG or BCG∆BCG1419c as adjuvants without infection vs. their infected counterpart groups, and (***) when both adjuvants were compared between them. Group I = not vaccinated/not infected; group II = not vaccinated/infected; group III = TcTPE-vaccinated + wtBCG as adjuvant/not infected; group IV = TcTPE-vaccinated + wtBCG as adjuvant/infected; group V = TcTPE-vaccinated + BCG∆BCG1419c as adjuvant/not infected; group VI = TcTPE-vaccinated + BCG∆BCG1419c as adjuvant/infected; group VII = TcTPE-vaccinated/not infected; group VIII = TcTPE-vaccinated/infected.

To assess the potential adverse effects of the vaccines, mice clinical parameters such as body weight, temperature, and physical condition were evaluated. Following booster vaccination, significant weight loss (* *p* ≤ 0.05) was observed in the groups that received vaccine formulations with any of the *M. bovis* BCG strains (groups III, IV, V, and VI), and this difference was accentuated in those that received the formulations with BCG Pasteur ATCC 35734 as the adjuvant (groups III and IV) (*** *p* ≤ 0.05) compared with group I (healthy). During the acute stage of infection, there was a significant weight reduction in almost all groups compared to group I, except for group VII, not-infected mice that were vaccinated only with the TcTPE formulation without any adjuvant; that is, those infected (groups II, IV, VI, and VIII) and even those not infected but who received the formulation containing either of two adjuvants (groups II and V) showed losses in their body weight. Groups IV and VI showed a significant difference (** *p* ≤ 0.05) against their healthy counterparts (groups III and V, respectively); these results indicate that the infection had synergy with the adjuvants in the weight loss of mice; in addition, the importance of the infection with *T. cruzi* in weight loss was confirmed, since in *T. cruzi*-challenging mice who had received only TcTPE (group VIII), the difference in weight reduction was greater compared with its not-infected counterparts (group VII) (Figure 3).

**Figure 4:**
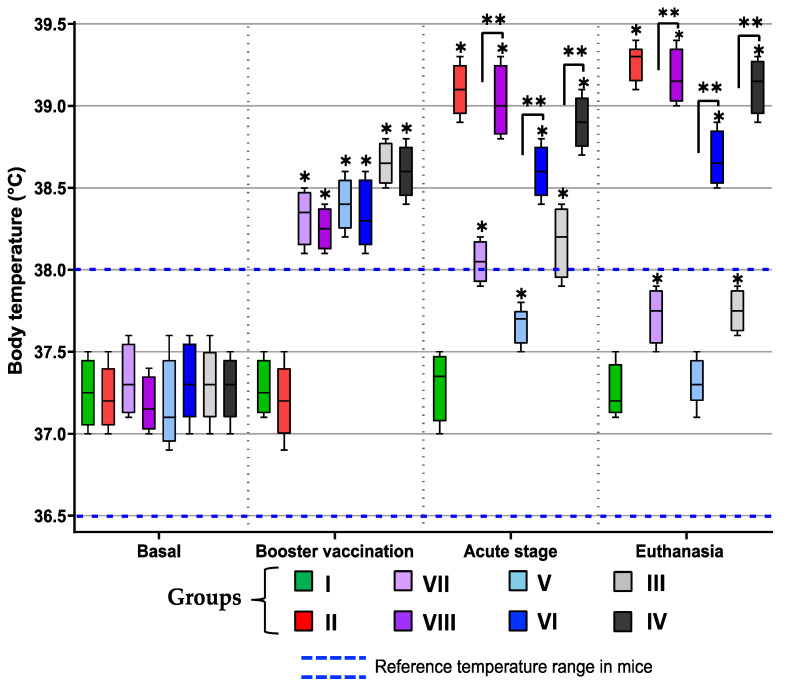
Body temperature of mice immunized with the vaccine formulation of TcTPE using *M. bovis* BCG strains as adjuvants and infected with *T. cruzi*. Values were analyzed with the one-way ANOVA statistical test followed by the Tukey post hoc test at each time point (basal, booster vaccination, acute stage, and euthanasia), and a significant difference was demonstrated (*, *p* ≤ 0.05) when comparing against the group I (healthy mice), and (**, *p* ≤ 0.05) when comparing the groups immunized with wtBCG or BCG∆BCG1419c as adjuvants without infection (groups III and V, respectively) vs. their infected counterpart groups (groups IV and VI, respectively). The dotted lines show the normal temperature range in mice. Group I = not vaccinated/not infected; group II = not vaccinated/infected; group III = TcTPE-vaccinated + wtBCG as adjuvant/not infected; group IV = TcTPE-vaccinated + wtBCG as adjuvant/infected; group V = TcTPE-vaccinated + BCG∆BCG1419c as adjuvant/not infected; group VI = TcTPE-vaccinated + BCG∆BCG1419c as adjuvant/infected; group VII = TcTPE-vaccinated/not infected; group VIII = TcTPE-vaccinated/infected.

Following the booster vaccination, the groups that received the immunogenic stimulus showed fever, as expected. In the acute stage of the ChD, the temperature remained high in all immunized groups infected or not (III, IV, V, VI, VII and VIII) compared to the healthy mice (group I and II before infection). In the course of the infection in group V, this effect was slight but within the reference values; not so for the rest of the groups—infected (II, IV, VI and VIII) or not (III and IV)—which recorded an increase in temperature above the values recorded post-vaccination, suggesting that the fever was a consequence of the disease, and not a vaccine-related side effect. The fever pattern remained constant until the time of euthanasia, unlike the immunized and not-infected groups (III, V and VII), which returned to the reference temperature range in mice, with group V showing an average temperature equal to that of group I (healthy mice) (Figure 4).

**Figure 5:**
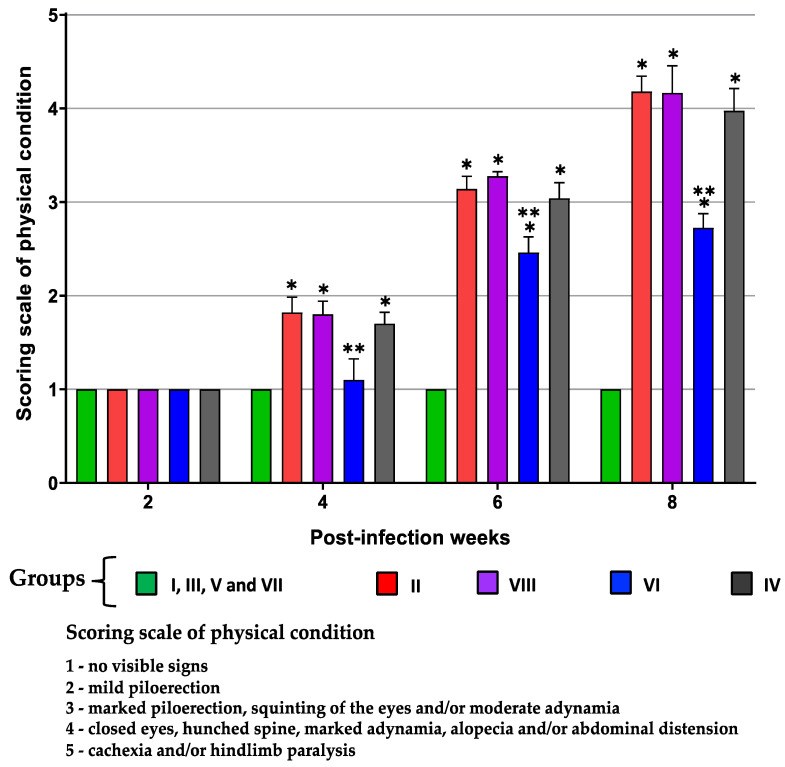
Physical condition record of mice immunized with the vaccine formulation of TcTPE using *M. bovis* BCG strains as adjuvants and infected with *T. cruzi*. Values show the mean with the S.D. of each group, which were analyzed with the nonparametric statistical test of Kruskal–Wallis followed by the Dunn post hoc test, and a significant difference was demonstrated (*, *p* ≤ 0.05) when comparing against the group I (healthy mice) and (**, *p* ≤ 0.05) when comparing against the group II (not vaccinated and infected mice). Group I = not vaccinated/not infected; group II = not vaccinated/infected; group III = TcTPE-vaccinated + wtBCG as adjuvant/not infected; group IV = TcTPE-vaccinated + wtBCG as adjuvant/infected; group V = TcTPE-vaccinated + BCG∆BCG1419c as adjuvant/not infected; group VI = TcTPE-vaccinated + BCG∆BCG1419c as adjuvant/infected; group VII = TcTPE-vaccinated/not infected; group VIII = TcTPE-vaccinated/infected.

The physical conditions of the mice were monitored for eight weeks to qualitatively assess their disease severity. It was observed that as the time of infection progressed, the score increased, corresponding to a visible deterioration in the mice’s health state. Two mice in group VIII (TcTPE-vaccinated/infected) reached a score of 5, which required applying euthanasia for humane reasons. A notable difference was recorded in animals from group VI (TcTPE + BCG∆BCG1419c vaccinated/infected), which did not show severe disease signs at any of the weeks evaluated, suggesting that the TcTPE vaccine formulation in combination with BCG∆BCG1419c as an adjuvant reduced some severe signs of overt ChD. Likewise, it was found that immunization per se did not have an effect on adverse clinical sign presentation (Figure 5).

### 3.3. Administration of Vaccine Induced Humoral Response

Levels of anti-*T. cruzi* IgG antibodies and their isotypes (IgG1 and IgG2a) induced during vaccination and after experimental infection were determined in order to examine the effect of *M. bovis* BCG strains as adjuvants.

**Figure 6:**
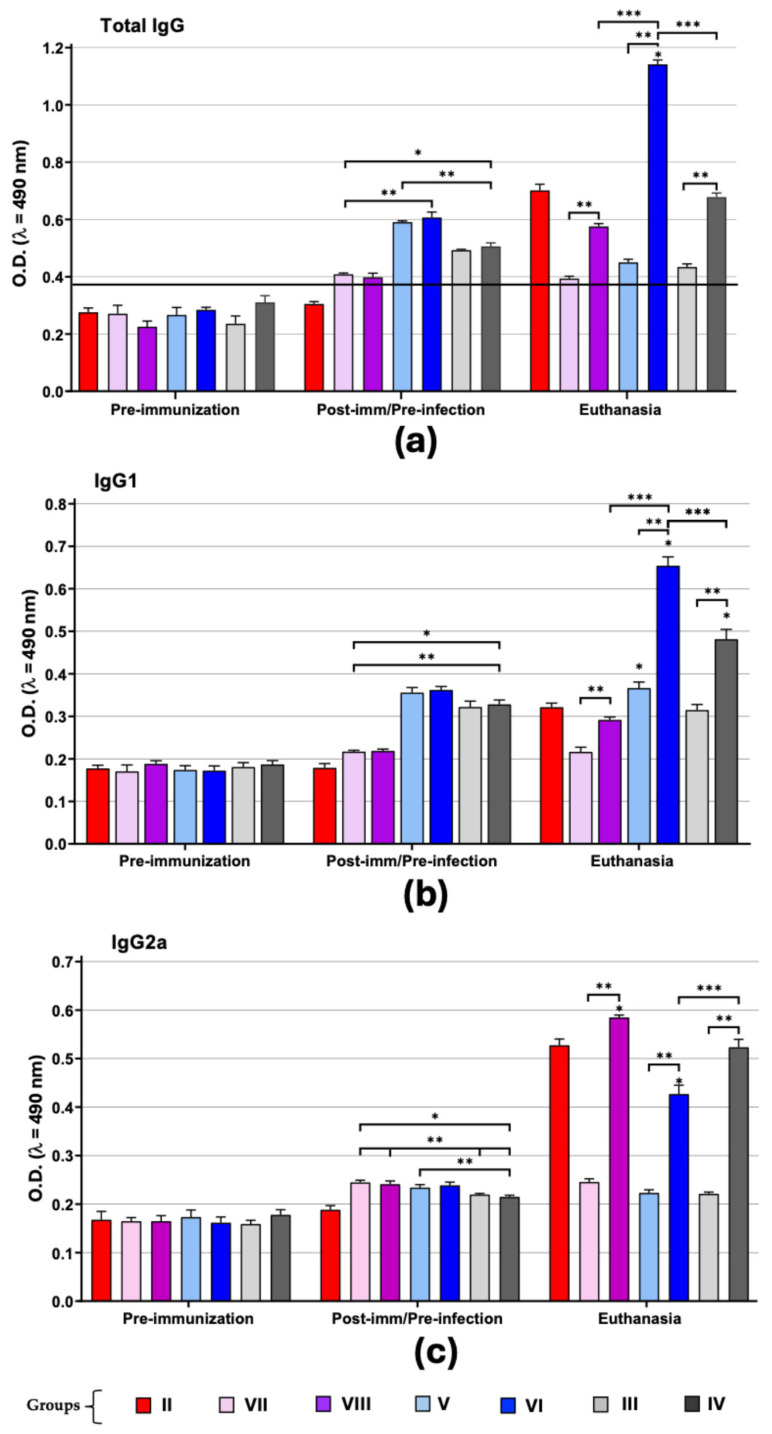
Humoral immune response in mice immunized with the vaccine formulation of TcTPE using *M. bovis* BCG strains as adjuvants and infected with *T. cruzi*. Values represent the mean ± S.D. of the optical densities (O.D.) of total IgG (**a**), IgG1 (**b**), and IgG2a (**c**) by group. Statistical analysis was performed using the one-way ANOVA test followed by the Tukey post hoc test. The results were considered significant when (*) *p* ≤ 0.05 when comparing against the II group infected mice, (**) *p* ≤ 0.05 when comparing the vaccinated group against its infected pair, and (***) *p* ≤ 0.05 when comparing the immunized/infected groups between them. Post-imm = post-immunization. The black line in (**a**) shows the cut-off value in the ELISA. Group I = not vaccinated/not infected; group II = not vaccinated/infected; group III = TcTPE-vaccinated + wtBCG as adjuvant/not infected; group IV = TcTPE-vaccinated + wtBCG as adjuvant/infected; group V = TcTPE-vaccinated + BCG∆BCG1419c as adjuvant/not infected; group VI = TcTPE-vaccinated + BCG∆BCG1419c as adjuvant/infected; group VII = TcTPE-vaccinated/not infected; group VIII = TcTPE-vaccinated/infected.

In the post-immunization/pre-infection period, all immunized groups induced the production of anti-*T. cruzi* IgG antibodies by the administration of TcTPE regardless of whether it was administered together with BCG∆BCG1419c or BCG Pasteur ATCC 35734 as an adjuvant. In the case of total IgG antibodies (Figure 6a), a higher production of anti-*T. cruzi* antibodies was induced in mice vaccinated with TcTPE plus BCG∆BCG1419c as an adjuvant (groups V and VI). The wtBCG strain had no adjuvant effect, since the induced IgG antibody titers were similar to those of animals vaccinated with the antigen alone. At the time of euthanasia, antibody production was significantly increased in previously vaccinated-infected animals, and these levels were not reached with immunization alone; however, the infection by itself elicited greater antibodies production (group II) than that achieved by vaccination/infection when the TcTPE (group VIII) and TcTPE plus wtBCG (group IV) vaccine formulations were used, and only group VI (TcTPE + BCG∆BCG1419c vaccinated/infected) showed a significant difference with respect to group II (* *p* ≤ 0.05) and with respect to the groups in which the other two vaccine formulations were administered (*** *p* ≤ 0.05), indicating that, during the acute stage, vaccination enhanced the production of anti-*T. cruzi* antibodies when BCG∆BCG1419c was used as an adjuvant.

It was confirmed that the two strains of BCG stimulated a specific immune response against *M. bovis* by determining the levels of anti-PPD antibodies (Supplementary data); it was also observed that the anti-PPD antibody profile was higher during the post-infection time, which suggested that *T. cruzi* infection had an immunostimulatory effect by increasing the immune response against *M. bovis* as previously reported when trained immunity was explored in experimental ChD in mice [9].

### 3.4. BCG Strains as Adjuvants Induced a Differential Immune Response by IgG1/IgG2a Ratio

When comparing the levels of IgG1 vs. IgG2a subclasses (Supplementary data), it was observed that immunization induces the production of higher levels of IgG1 than IgG2a in groups V and VI (TcTPE + BCG∆BCG1419c-vaccinated mice) (Figure 6b,c). In these groups (V and VI), the stimulation of the vaccine formulation caused a slight polarization (less than double) to a Th2 immune profile favoring an anti-inflammatory environment, which, for the *T. cruzi* infection (group VI), this response persisted at an average of 1.5 ratio during all acute phase of ChD. These data suggest that this vaccine formulation (groups V and VI) conferred a differential immune response (*** *p* ≤ 0.05) with respect to the other groups that helps counteract the inflammatory environment stimulated by the production of IgG2a > IgG1 that has always been described in an acute infection by *T. cruzi*.

In contrast, the stimulation with wtBCG-adjuvanted vaccine formulation (groups III and IV) initially promoted a slight polarization of less than 1.5 times toward a Th1 immune profile, favoring an inflammatory response. When *T. cruzi* infection occurred, the host tried to restrain the inflammatory environment that was being generated by a mixed Th1/Th2 immune response of similar proportions (1.09 ratio); however, only 20% of the mice showed an improvement in their physical condition, while 80% died.

### 3.5. Vaccine Formulation Using BCGΔBCG1419c as an Adjuvant Induced a Th1/Th2 Balanced Immune Response by the Production of Serum Cytokines

**Figure 7:**
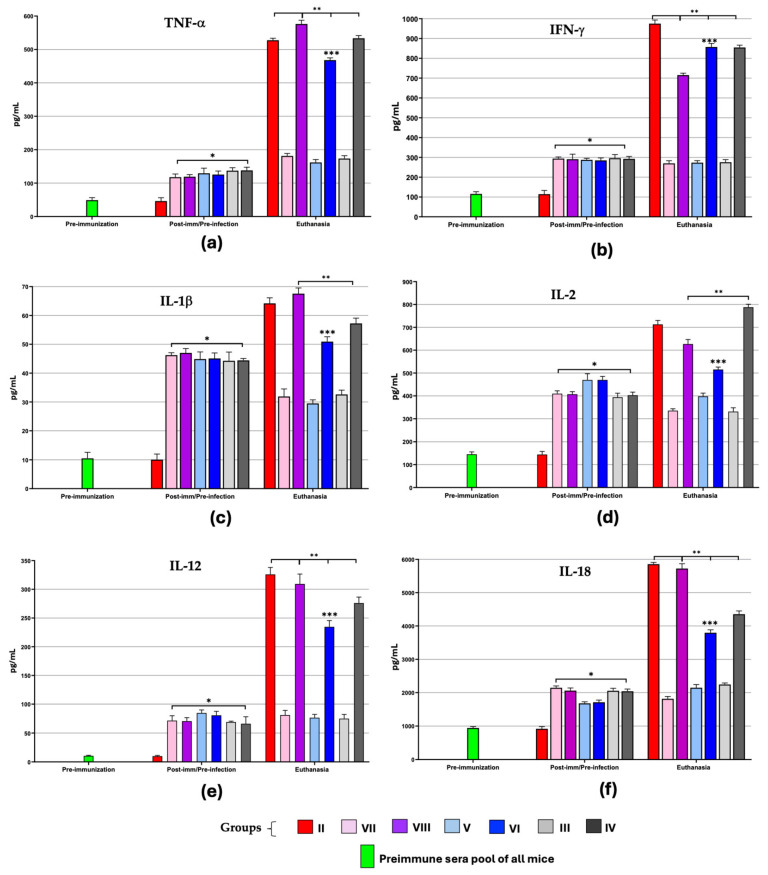
Serum cytokines concentration of the Th1 immune profile in mice immunized with the vaccine formulation of TcTPE using *M. bovis* BCG strains as adjuvants and infected with *T. cruzi.* Values represent the mean ± S.D. of the cytokine levels (pg/mL) by group: TNF-α (**a**), IFN-γ (**b**), IL-1β (**c**), IL-2 (**d**), IL-12 (**e**), and IL-18 (**f**). The one-way ANOVA statistical test followed by the Tukey post hoc test was used to determine the existence of a significant difference (* *p* ≤ 0.05) with respect to the baseline concentration (preimmune sera pool of all mice or group II at post-imm/pre-infection time), (** *p* ≤ 0.05) of the euthanasia-time groups with respect to their corresponding ones in the post-vaccination/pre-infection time and (*** *p* ≤ 0.05) when the infected groups (vaccinated or not) were compared between them. Post-imm = post-immunization. Group I = not vaccinated/not infected; group II = not vaccinated/infected; group III = TcTPE-vaccinated + wtBCG as adjuvant/not infected; group IV = TcTPE-vaccinated + wtBCG as adjuvant/infected; group V = TcTPE-vaccinated + BCG∆BCG1419c as adjuvant/not infected; group VI = TcTPE-vaccinated + BCG∆BCG1419c as adjuvant/infected; group VII = TcTPE-vaccinated/not infected; group VIII = TcTPE-vaccinated/infected.

**Figure 8:**
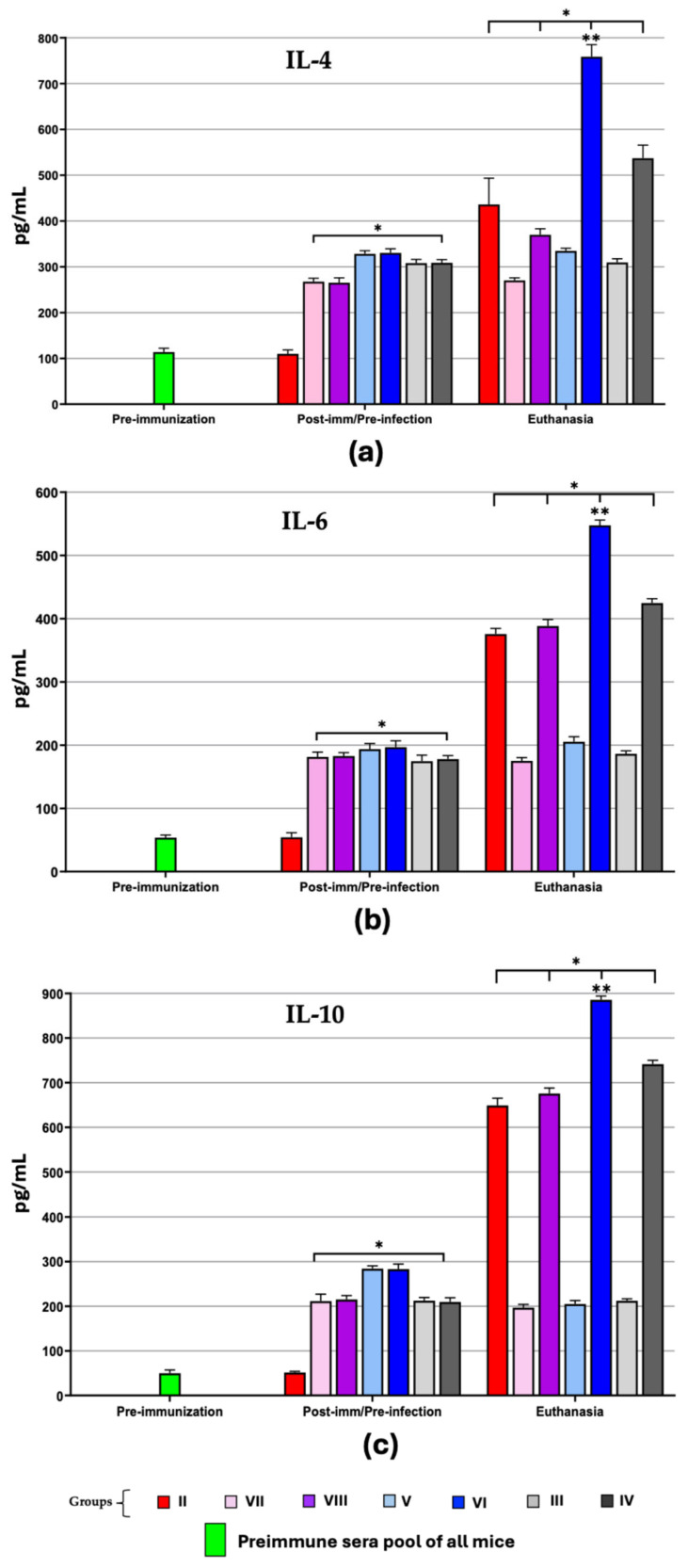
Serum cytokines concentration of the Th2 immune profile in mice immunized with the vaccine formulation of TcTPE using *M. bovis* BCG strains as adjuvants and infected with *T. cruzi.* Values represent the mean ± S.D. of the cytokine levels (pg/mL) by group: IL-4 (**a**), IL-6 (**b**), and IL-10 (**c**). One-way ANOVA statistical test followed by Tukey’s post hoc test was used to determine significant difference (* *p* ≤ 0.05) with respect to the baseline concentration (preimmune sera pool of all mice or group II at post-imm/pre-infection time) and (** *p* ≤ 0.05) when comparing the infected groups (vaccinated or not) among them. Post-imm = post-immunization. Group I = not vaccinated/not infected; group II = not vaccinated/infected; group III = TcTPE-vaccinated + wtBCG as adjuvant/not infected; group IV = TcTPE-vaccinated + wtBCG as adjuvant/infected; group V = TcTPE-vaccinated + BCG∆BCG1419c as adjuvant/not infected; group VI = TcTPE-vaccinated + BCG∆BCG1419c as adjuvant/infected; group VII = TcTPE-vaccinated/not infected; group VIII = TcTPE-vaccinated/infected.

The cellular immune response of ChD was evaluated by determining some serum cytokines, six main ones of a Th1 immune profile (TNF-α, IFN-γ, IL-1β, IL-2, IL-12, and IL-18) (Figure 7) and three Th2 ones (IL-4, IL-6, and IL-10) (Figure 8), to assess which vaccine formulation best controls the inflammatory environment induced by the parasite and, therefore, helps mice to show an improvement against progression in the lethal disease.

In post-immunization/pre-infection time, all vaccinated groups showed an increase (* *p* ≤ 0.05) in the TNF-α, IFN-γ, IL-1β, IL-2, IL-12, and IL-18 concentrations with no difference between them. These results indicate that the three vaccine formulations stimulated the production of Th1-type immune profile cytokines (Figure 7). They also show that the use of any of the BCG strains as adjuvants did not modify the levels of these cytokines induced by the TcTPE antigenic stimulus. At the time of euthanasia (ChD acute phase), a statistically significant increase (** *p* ≤ 0.05) was recorded in TNF-α, IFN-γ, IL-12, and IL-18 concentrations in only-infected groups, immunized, or not immunized (groups II, IV, VI and VIII); while in the groups that did not have the stimulus produced by the parasite infection (groups III, V, and VII), levels of these cytokines remained similar to those during post-immunization/pre-infection time (*p* > 0.05).

In order to determine whether the BCG strains used as adjuvants induced a differential polarization of the immune response compared to the rest of the groups, the IFN-γ/IL-4 ratio was calculated (supplementary data). In TcTPE + BCG∆BCG1419c-vaccinated mice (groups V and VI) the stimulation of the vaccine formulation did not cause predominance of any of these two cytokines (1.16–1.24 ratios, respectively). Therefore, it can be inferred that BCG∆BCG1419c strain as adjuvant induced a Th1/Th2 balanced or mixed immune response, which is consistent with what was observed in the production of IgG1/IgG2a levels. Also, it is important to highlight that only group VI (TcTPE + BCG∆BCG1419c-vaccinated/infected mice) had low levels (*** *p* ≤ 0.05) of all the inflammatory cytokines tested with respect to the rest of the immunized or non-immunized/infected mice, such that these levels were sufficient to control the progression of ChD without causing the death of the animals in the acute phase, thus suggesting that this formulation adequately protected against ChD.

On the other hand, no polarization towards any response profile was observed with wtBCG-adjuvanted vaccine formulation (groups III and IV), and there was a slight polarization towards the Th1-type inflammatory response only when acute infection was present (group IV), which was probably the cause of some of the animals’ deaths, consistent also with a significant increase (** *p* ≤ 0.05) in IL-1β and IL-2 concentrations at the time of euthanasia vs. post-immunization/pre-infection time.

In post-vaccination/pre-infection time, all immunized groups showed a significant increase (* *p* ≤ 0.05) of IL-4, IL-6, and IL-10 cytokines with 200–300 pg/mL levels, with no difference between groups, very similar to the TNF-α, IFN-γ, and IL-12 concentrations but lower than IL-2 and IL-18 and higher than IL-1β levels; therefore, it is not possible to determine with certainty if there is a predominance of any immunological profile at this time (Figure 8). At the time of euthanasia (acute stage of *T. cruzi* infection), IL-4, IL-6, and IL-10 levels had a small increase in the four groups that were infected (three immunized and one was not), while in the immunized but not-infected groups, concentrations remained similar to those at the post-immunization/pre-infection time (*p* > 0.05).

### 3.6. Tissues from Mice Vaccinated with the BCGΔBCG1419c Formulation as an Adjuvant Showed Less Damage in Heart and Skeletal Muscle

In the acute stage, at 30th dpi, when the highest number of deaths occurred in groups II, IV, and VII, all animals were euthanized for the evaluation of macro- and microscopic findings. To determine visceral megalia, the index of the heart, spleen, and lymph nodes was calculated, and no differences were found with respect to the group I (healthy mice), except for cardiomegaly and splenomegaly in the group II (Supplementary data).

**Figure 9:**
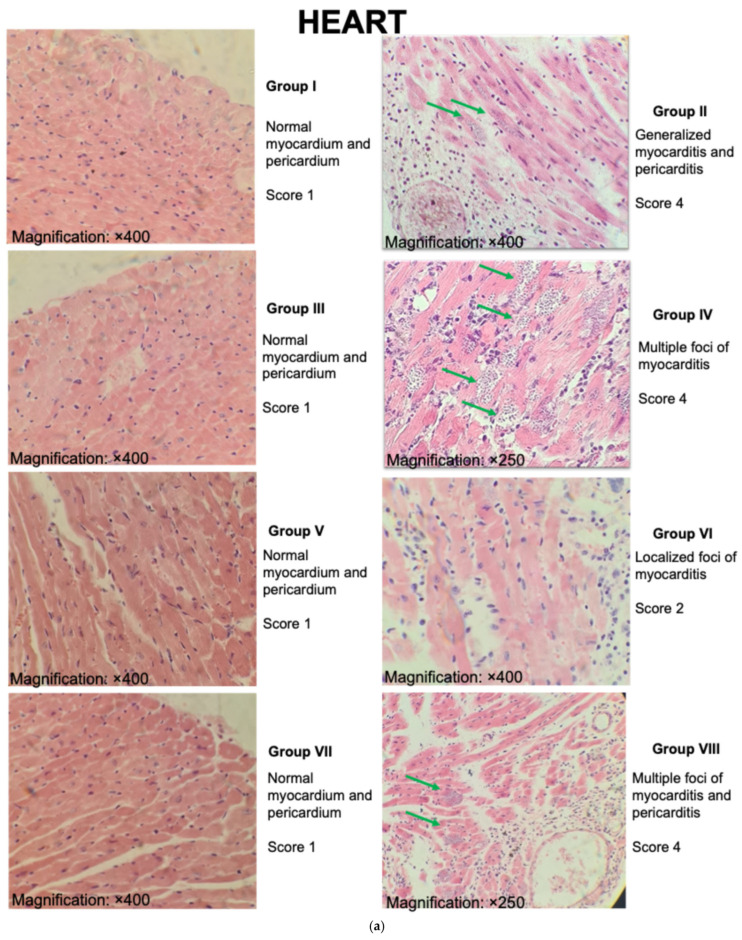

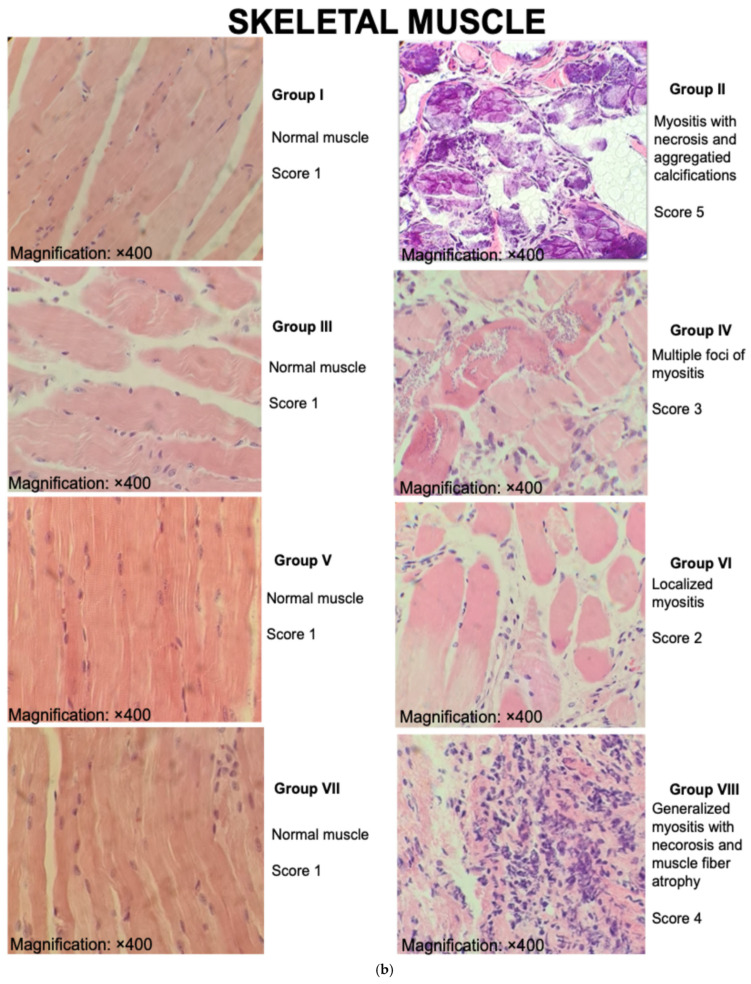
Histopathological findings in tissue samples of heart (**a**) and skeletal muscle, (**b**) from mice immunized with the vaccine formulation of TcTPE using *M. bovis* BCG strains as adjuvants and infected with *T. cruzi*. Representative micrographs of each experimental group are shown. Green arrows indicate amastigotes nests. Group I = not vaccinated/not infected; group II = not vaccinated/infected; group III = TcTPE-vaccinated + wtBCG as adjuvant/not infected; group IV = TcTPE-vaccinated + wtBCG as adjuvant/infected; group V = TcTPE-vaccinated + BCG∆BCG1419c as adjuvant/not infected; group VI = TcTPE-vaccinated + BCG∆BCG1419c as adjuvant/infected; group VII = TcTPE-vaccinated/not infected; group VIII = TcTPE-vaccinated/infected. Magnification: ×250 or ×400.

**Figure 10:**
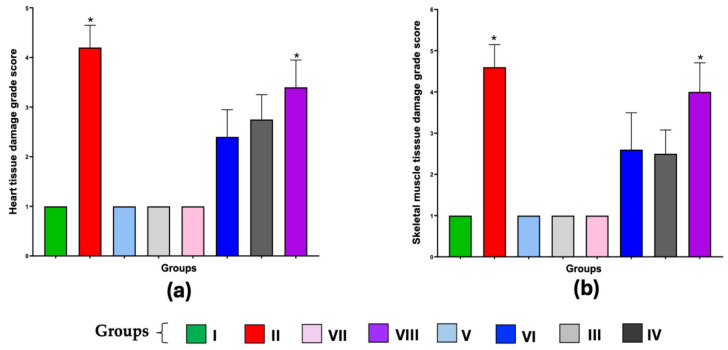
Scores of tissue damage degree from mice immunized with the vaccine formulation of TcTPE using *M. bovis* BCG strains as adjuvants and infected with *T. cruzi.* Values represent the mean ± S.D. of the heart (**a**) or skeletal muscle, (**b**) damage degree scores from each mouse per group at 30th dpi (acute phase of ChD). Kruskal–Wallis test was used for determining significant differences when (*) *p* ≤ 0.05 when comparing all groups with the I group healthy mice. Group I = not vaccinated/not infected; group II = not vaccinated/infected; group III = TcTPE-vaccinated + wtBCG as adjuvant/not infected; group IV = TcTPE-vaccinated + wtBCG as adjuvant/infected; group V = TcTPE-vaccinated + BCG∆BCG1419c as adjuvant/not infected; group VI = TcTPE-vaccinated + BCG∆BCG1419c as adjuvant/infected; group VII = TcTPE-vaccinated/not infected; group VIII = TcTPE-vaccinated/infected.

Cardiac and skeletal muscle tissue were analyzed histopathologically, findings were represented quantitatively, and the values were compared with those of the group I with a score of 1 (healthy mice). The group immunized with TcTPE using BCG∆BCG1419c as an adjuvant and infected with *T. cruzi* (Group VI) showed minor histological changes, with a damage grade score of 2 in both cardiac tissue and skeletal muscle. Group VIII, as well as group II, in which animals died due to ChD, presented the highest number of tissue amastigotes nests with a damage grade score greater than 4 in cardiac muscle and skeletal muscle. The tissue damage score did not represent a significant difference in group IV (TcTPE + wtBCG-vaccinated/infected); however, 80% of the animals died, and all of them (100%) had marked physical deterioration (Figure 9 and Figure 10).

Analysis of the tissues of the infected groups showed that the group immunized using the BCG∆BCG1419c adjuvant showed a degree of damage of 2 points for the cardiac muscle and 3 points for the skeletal muscle, demonstrating a protective effect against ChD.

## 4. Discussion

Monocomponent (single antigen) vaccines have shown partial success in eradicating *T. cruzi*; consequently, the idea of combining different antigens that previously functioned as candidate vaccines with adjuvant agents to increase the efficacy of immune response arose more than a decade ago [22]. In this study, the efficacy in reducing parasitemia and mortality was observed with better parameters when vaccination with TcTPE and BCG∆BCG1419c was used as an adjuvant, with a reduction in parasitemia of 50.32% and with a mortality rate only of 20%; in contrast, when the wtBCG strain was used as an adjuvant, its parasitemia reduction was only 35% and the mortality rate was 80%. Frank et al. (2003) used purified *T. cruzi* antigen and CpG oligodeoxynucleotides as an adjuvant to formulate a vaccine, which was administered to inbred C3H/HeN-strain mice that were subsequently infected with 500 blood trypomastigotes of the RA strain [23]; they observed that the vaccine significantly reduced parasitemia by approximately 60%; likewise, 100% of the group survived the infection. Additionally, they determined that immunization with only the purified *T. cruzi* antigen did not significantly reduce parasitemia and showed a survival rate of 0%. These authors’ results resemble those presented here. It has been reported that SC immunization with recombinant *T. cruzi* paraflagellar protein in BALB/c mice subcutaneously infected with 10^2^ blood trypomastigotes of the Peru strain reduced parasitemia by up to 70% and produced 100% survival [24]. Another report explains the design of a mutant of the transialidase enzyme as a potential vaccine, which was administered to BALB/c mice that were subsequently infected with 100 blood trypomastigotes of the Tulahuén strain; the results showed a significant reduction in parasitemia of 100% and a survival rate of 100% [25]. Studies with recombinant or mutant proteins report better results than those obtained with the TcTPE used in the present study. Recently, recombinant *M. bovis* BCG expressing NT-TS antigens specific to *T. cruzi* (amino-N and carboxyl-C fragments of the enzyme transialidase, and a fragment of the cruzipain enzyme) in pUS2000 plasmid administered to BALB/c mice that were subsequently infected with 500 blood trypomastigotes of the Tulahuén strain showed a 55% reduction in parasitemia and a survival rate of 80% [26]. These results are very similar to those obtained here with vaccination using BCG∆BCG1419c as an adjuvant.

A significant reduction in body weight was registered in mice immunized with BCG∆BCG1419c (30.3%) or BCG Pasteur ATCC 35734 (68.8%) as adjuvants, which is probably attributed to the presence of the bacillus [27]. Other authors observed that only male BALB/c mice vaccinated with *M. bovis* Danish 1331 BCG strain (BCG SSI) showed a significant weight loss of 20% [28]; however, in the present study females BALB/c showed such effect with *M. bovis* Pasteur ATCC 35734 BCG strain. Immunized and infected mice with a vaccine based on a mutation in transialidase had normal weights, while the infected, not vaccinated mice lost significant weight [25], which is attributed to the infection, as in the present study, but it was also evident that BCG∆BCG1419c or BCG Pasteur ATCC 35734 as adjuvants contributed to a greater decrease in weight, demonstrating that the use of adjuvants has many other implications on the health status of animals.

Fever, as a general natural effect of every vaccine [29], was observed in all TcTPE-vaccinated groups, with and without any BCG adjuvant, during the post-vaccination/pre-infection period, indicating that the immune system of the mice responded against immunization; subsequently, the recorded temperature returned to the reference values. Only the mice in group II (not vaccinated/infected) showed an increase in temperature at post-infection time, a classic sign of acute ChD [30]. In the acute stage of ChD, group VI (TcTPE + BCG∆BCG1419c-vaccinated/infected mice) had a lower body temperature than the other groups; this may be related to the control of parasitemia, since this group showed a lower parasitic load. On the other hand, in a ChD model in Rhesus macaques (*Macaca mulatta*) vaccinated with recombinant *T. cruzi* proteins such as trypomastigote surface antigen (TSA-1) and calcium-binding flagellar antigens (Tc24), no fever was recorded after vaccination with the antigens in animals without *T. cruzi* infection [31].

The animals’ physical condition was not affected by the administration of TcTPE vaccines using wtBCG or BCG∆BCG1419c as adjuvants, showing a score of 1 (no visible signs). This result is comparable with that obtained by others in the Rhesus macaque model of ChD, in which no changes in general health were reported with *T. cruzi* protein antigen vaccines [31]. Other authors have evaluated certain clinical parameters of ChD in mice infected with *T. cruzi* Y strain, reporting relevant alterations in parameters such as water and food consumption, stool characteristics, body temperature, abdominal circumference, mobility, weight, and mortality [32]. A similar scoring scale to the one used here was employed by others [28], and they observed no changes in the physical condition of BALB/C mice vaccinated with *M. bovis* BCG against tuberculosis, which is consistent with what was obtained with TcTPE vaccine formulations against *T. cruzi* with BCG strains as adjuvants. In the canine model of ChD with Beagle dogs, it was previously reported that immunized animals showed fewer physical signs than unvaccinated animals [33]; these results were similar to those observed in the present study.

It was observed that vaccination stimulated significant IgG antibody production in group VI (TcTPE + BCG∆BCG1419c-vaccinated/infected mice) compared to groups IV and VIII (TcTPE + wtBCG-vaccinated/infected and TcTPE-vaccinated/infected mice, respectively), which showed similar levels to group II (not vaccinated/infected mice), this result is similar to that observed in a study in which BALB/c mice were subcutaneously vaccinated with a glycosylated mutant transialidase as antigen and the ISCOMATRIX complex—compound made of some saponins and a matrix of cholesterol- and phospholipid-based nanoparticles—as an adjuvant, and then infected intraperitoneally with 1000 blood trypomastigotes from the *T. cruzi* Tulahuén strain [34]; the authors observed an effective humoral response in the infected group that received the vaccine, since it showed a high IgG antibody titer four times higher than the group that was vaccinated with recombinant transialidase alone, which denotes the importance of having a good adjuvant. When administered recombinant *M. bovis* BCG expressing NT-TS antigens specific to *T. cruzi*, a higher level of IgG antibodies with respect to the infected control group and the groups vaccinated only with the *M. bovis* BCG as an adjuvant was observed [26]. The choice of an appropriate adjuvant together with antigens that are capable of mounting a memory response can enhance the production of specific IgG antibodies despite the ongoing infection and, therefore, contribute to the clearance of parasites and consequent protection against the symptoms of the disease.

We observed that in group VI (TcTPE + BCG∆BCG1419c-vaccinated/infected mice) there was a slight polarization (less than double) bias towards a Th2 or anti-inflammatory response, where such a response persisted in an average IgG1/IgG2a ratio of 1.5 during all the acute phases of ChD. This immunological profile has been described to lead to a better prognosis in chronic infection by modulating the Th1 response to control cardiac inflammation and avoid damage to important tissues that lead to mortality [35,36].

Groups II, IV, and VIII showed higher levels of Th1-profile cytokines and lower concentrations of Th2, demonstrating polarization towards a pro-inflammatory response, which, although important for eradicating the parasite in the acute stage through the cytotoxic effector function of this type defense system stimulated by *T. cruzi* as a facultative intracellular pathogen [37], high concentrations of these cytokines could harm the organism and cause mortality as the disease progresses to the chronic phase, which was observed in these groups. TNF-α, IFN-γ, IL-12, and IL-18 increasing could be related to the activity of macrophages in response to the presence of *T. cruzi*, and by positive feedback of these same cytokines on NK cells [38]. Mortality in these groups during the acute stage can be explained by the fact that the cytokine IL-1β is involved in the overproduction of nitric oxide (NO) and monocytes when IL-6 levels are deficient; in addition, IL-2 is related to the proliferation of CD8+ T lymphocytes, cells involved in the control of parasites, and are associated with myocarditis. Therefore, these cytokines must be adequately regulated for efficient control of the disease because at high concentrations they can cause early mortality. On the other hand, the main function of IL-4 is to suppress the production of IFN-γ and NO, while IL-10 decreases the synthesis of TNF-α and IFN-γ.

On the other hand, IL-6 modulates the inflammation induced by CHD by inhibiting the production of TNF-α, IL-1β, and IL-1. Control over inflammatory cytokines is related to their contribution to tissue and organ damage that can trigger early mortality; therefore, IL-4, IL-6, and IL-10 in the acute stage of ChD control the inflammatory response and allow development to the asymptomatic chronic stage of the disease [38].

BCG∆BCG1419c turned out to be a good adjuvant like other bacterial agents [39,40], since it achieved a reduction in parasitic load and tissue damage in target organs. Inflammatory infiltrate and splenomegaly are frequent findings in the tissues of individuals infected with *T. cruzi*, regardless of parasite strain or host [41]; in the present study this was corroborated, and by using BCG∆BCG1419c as a vaccine adjuvant, these alterations decreased.

Due to the mortality that occurs during the acute phase of ChD in this model in not vaccinated and/or untreated animals, a prospective study should be able to analyze the same parameters in the chronic phase; chronic-stage data would help determine whether vaccination offers long-term protection by preventing or delaying the development of cardiomyopathy. In this sense, it is acknowledged that the use of female mice is a limitation to the potential translational impact of our current findings; however, the design focused on ensuring the establishment of a progressive and non-lethal infection, which is better achieved with females than with males; furthermore, our using females here is consistent with previous studies in which the wild-type BCG strain was used as an immunostimulant. Regarding future research, we endeavor to conduct the evaluation of efficacy at the chronic stage of ChD, in which populations of both sexes can be included. To attain this, it is necessary to use less virulent *T. cruzi* strains or to perform tests with *T. cruzi* sub-lethal infective doses, which is difficult to achieve, and this is a limitation in this study, since the few parasites used for infection can often be eliminated by the innate immune systems of some individuals. Despite having ensured reproducibility through two independent experiments, the small sample size tested here remains a technical limitation. Consequently, we acknowledge that to suggest extrapolation of our findings to a larger population requires further examination. It should also be noted that the study is constrained by the use of a crude extract in which the nature of the predominant antigen(s) in the stimulation of the immune response is unknown. Therefore, future studies should use specific antigenic proteins already tested by our research group in order to have defined antigens that can provide further insights. Another limitation at the translational level is that two vaccination doses were used in this model, and the WHO currently recommends giving only one dose of BCG to infants and young children in countries where tuberculosis is common; therefore, future studies using BCG as an adjuvant against ChD could consider a single dose. At the same time, countries with more ChD have probably already received BCG at birth, which is another potential limitation to recognize in this research, although it opens up the possibility of assessing a regime where BCG∆BCG1419c and a *Trypanosoma cruzi* vaccine could be applied simultaneously. Finally, we acknowledge that no mechanistic insights were provided on the reasons for improved protection observed upon administration of BCG∆BCG1419c, and this is a caveat of this work and should be evaluated in a future study. Because of this, it has not been conclusively demonstrated whether the role of this mutant strain was promoting antigen-specific immunity or, rather, trained innate immunity; therefore, the effect of BCG∆BCG1419c alone could be evaluated in future research to examine its effect on ChD. Despite this, we contend that showing an improvement on the course of ChD upon administration of BCG∆BCG1419c is a valuable contribution for preclinical studies of vaccines against ChD that should be shared with the scientific community and is worthy of further characterization.

## 5. Conclusions

Having shown that the vaccine formulation composed of TcTPE plus BCGDBCG1419c provides protection during the acute stage of Chagas disease, this confirms it as a promising vaccine candidate, we endeavor to conduct further investigation to assess its efficacy against the chronic stage of this disease to determine its potential breadth of utilization against ChD.

## Figures and Tables

**Table 1:** Description of the experimental groups.

Group (*n* = 5)	TcTPE Vaccine (10–15 mg)	BCG Strain as Adjuvant (10^4^ CFU/Mouse)	Infection * (250 Blood Trypomastigotes/Mouse)
I	No	None	No
II	No	None	Yes
III	Yes	*M. bovis* BCG Pasteur strain ATCC 35734	No
IV	Yes	*M. bovis* BCG Pasteur strain ATCC 35734	Yes
V	Yes	BCG∆BCG1419c mutant strain derived from *M. bovis* BCG Pasteur strain ATCC 35734	No
VI	Yes	BCG∆BCG1419c mutant strain derived from *M. bovis* BCG Pasteur strain ATCC 35734	Yes
VII	Yes	None	No
VIII	Yes	None	Yes

* Ninoa *T. cruzi* strain (MHOM/MX/1994/Ninoa), CFU = colony-forming units.

**Table 2:** Histological damage grade score.

Damage Grade Score	Description
1	No histological alteration (healthy tissue)
2	Localized myocarditis/myositis
3	Multiple foci of myocarditis/myositis, localized calcifications, preserved tissue integrity
4	Pericarditis with generalized myocarditis/myositis, multiple calcifications, localized basophilic necrosis, moderately preserved tissue integrity
5	Pericarditis with generalized myocarditis/myositis, multiple calcifications, generalized basophilic necrosis, tissue integrity completely altered

**Table 3:** Means of blood trypomastigotes/mL of blood in different infected groups at the peak of parasitemia (28th–30th dpi).

Group (*n* = 5)	TcTPE Vaccine	BCG Strain as Adjuvant	Mean of BT/mL of Blood at Peak of Parasitemia	*p* ^1^
I	No	None	Not infected	N/A
II	No	None	4.9 × 10^6^	N/A
III	Yes	*M. bovis* BCG Pasteur strain ATCC 35734	Not infected	N/A
IV	Yes	*M. bovis* BCG Pasteur strain ATCC 35734	3.19 × 10^6^	* 0.0019
V	Yes	BCG∆BCG1419c mutant strain derived from *M. bovis* BCG Pasteur strain ATCC 35734	Not infected	N/A
VI	Yes	BCG∆BCG1419c mutant strain derived from *M. bovis* BCG Pasteur strain ATCC 35734	2.44 × 10^6^	* 0.0001 ** 0.007
VII	Yes	None	Not infected	N/A
VIII	Yes	None	5.26 × 10^6^	>0.05

^1^ Statistical significance. * Comparing with Group II (*T. cruzi*-infected, unvaccinated mice). ** Comparing Groups IV and VI with each other. TcTPE = total protein extract from *T. cruzi*, BT = blood trypomastigotes, N/A = not applicable.

## Data Availability

The original contributions presented in this study are included in the article/Supplementary Material. Further inquiries can be directed to the corresponding authors.

## Supplementary Materials

The following supporting information can be downloaded at https://www.mdpi.com/article/10.3390/microorganisms13112447/s1, Figure S1: Anti-PPD IgG antibody levels in mice immunized with the vaccine formulation of TcTPE using *M. bovis* BCG strains as adjuvants and infected with *T. cruzi*; Figure S2: Heart (a), splenic (b), and lymph node (c) indices with the vaccine formulation of TcTPE using *M. bovis* BCG strains as adjuvants and infected with *T. cruzi*; Table S1: Subclasses IgG2a/IgG1 or IgG1/IgG2a ratios; Table S2: IFN-γ/IL-4 or IL-4/ IFN-γ ratios.

## Abbreviations

The following abbreviations are used in this manuscript:

BT	Blood trypomastigotes
c-di-cGMP	Cyclic diguanylate
CDC	Centers for Disease Control and Prevention
CFU	Colony-forming units
ChD	Chagas disease
d	Days
dpi	Day post-infection
IL	Interleukin
IP	Intraperitoneal
IFN	Interferon
LIT	Liver Infusion Tryptose
MST	Median survival time
PBS	Phosphate-buffered solution
PPD	Purified protein derivative
SC	Subcutaneous
S.D.	Standard deviation
TcTPE	Total protein extract from *Trypanosoma cruzi*
TNF	Tumor necrosis factor
WHO	World Health Organization
wtBCG	*M. bovis* BCG Pasteur ATCC 35734 (wild-type BCG strain)
