# Supplementary material for: Vaccination with a Trypanosoma cruzi Protein Extract Plus BCG∆BCG1419c Promotes a Balanced Th1/Th2 Immune Profile That Improves Control of Acute Chagas Disease in BALB/c Mice
Source: Microorganisms. 2025 Oct 25;13(11):2447. doi: 10.3390/microorganisms13112447 (PMC12654836; doi:10.3390/microorganisms13112447)
Supplement: Supplementary file 1 [file microorganisms-13-02447-s001.zip › microorganisms-3895898-supplementary.pdf]

**Supplementary material**

Table of contents:


| Supplementary data | Page |
|---|---|
| Figure S1. Anti-PPD IgG antibody levels in mice immunized with the vaccine formulation of TcTPE using *M. bovis* BCG strains as adjuvants and infected with *T. cruzi.* | 2 |
| Table S1. Subclasses IgG2a/IgG1 or IgG1/IgG2a ratios. | 3 |
| Table S2. IFN-γ/IL-4 ratio. | 4 |
| Figure S2. Heart (a), splenic (b) and lymph node (c) indices with the vaccine formulation of TcTPE using *M. bovis* BCG strains as adjuvants and infected with *T. cruzi*. | 5 |

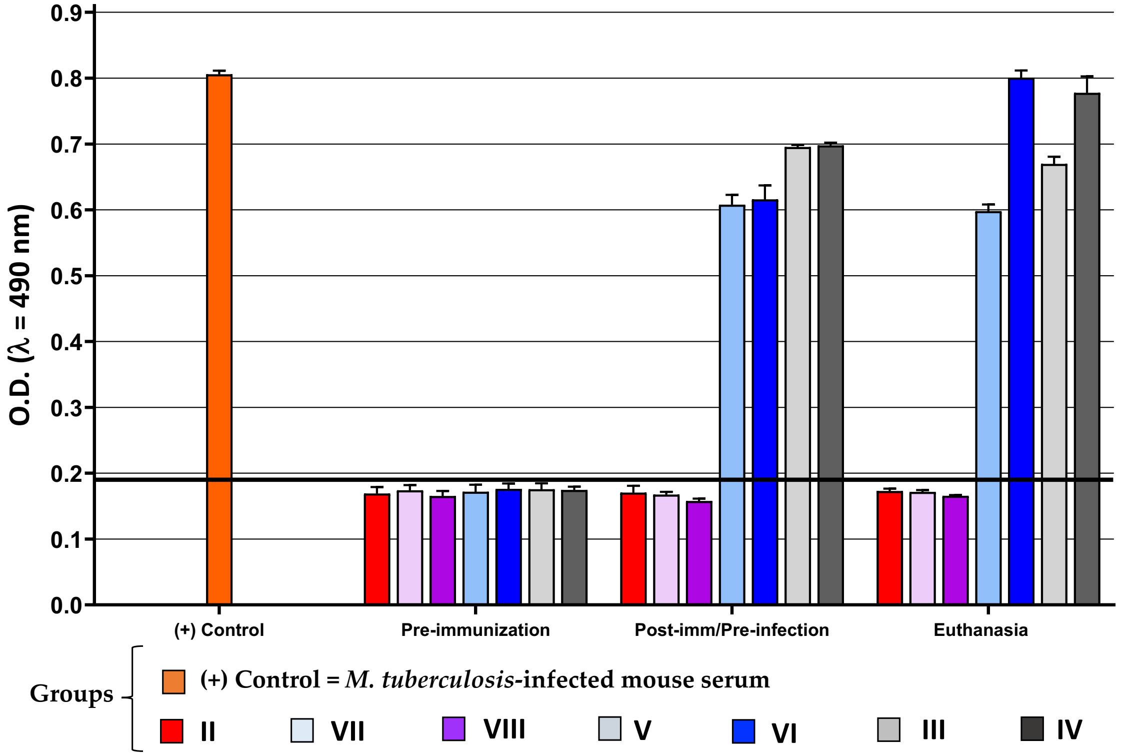


Figure S1. Anti-PPD IgG antibody levels in mice immunized with the vaccine formulation of TcTPE using *M. bovis* BCG strains as adjuvants and infected with *T. cruzi*. Values represent the mean ± S.D. of the optical densities (OD) of total IgG by group and are representative of two independent experiments with equivalent results. The black line corresponds to the cut-off value. Group II = not vaccinated/infected; group III = TcTPE-vaccinated+wtBCG as adjuvant/not infected; group IV = TcTPE-vaccinated+wtBCG as adjuvant/infected; group V = TcTPE-vaccinated+BCGΔBCG1419c as adjuvant/not infected; group VI = TcTPE-vaccinated+BCGΔBCG1419c as adjuvant/infected; group VII = TcTPE-vaccinated/not infected; group VIII = TcTPE-vaccinated/infected.

## Table S1. Subclasses IgG2a/IgG1 or IgG1/IgG2a ratios

| IgG2a/IgG1 ratio | | | |
|---|---|---|---|
| Time | Tc (Group II) | | |
| | IgG2a | IgG1 | Ratio |
| Postvac/Preinf | 0.188 | 0.1788 | 1.05 |
| Euth (acute phase) | 0.5272 | 0.3213 | **1.64** |

In group II, only in euthanasia (acute phase of ChD) we observed a Th1 polarization or inflammatory response in a 1.6 ratio, as *T. cruzi* infection has always been described at this stage of the disease.

| IgG2a/IgG1 ratio | | | | | | | |
|---|---|---|---|---|---|---|---|
| Time | TcTPE (Group VII) | | | Time | TcTPE/Tc (Group VIII) | | |
| | IgG2a | IgG1 | Ratio | | IgG2a | IgG1 | Ratio |
| Postvac/Preinf | 0.2445 | 0.21675 | 1.13 | Postvac/Preinf | 0.241 | 0.2185 | 1.10 |
| Euthanasia time | 0.2455 | 0.21625 | 1.14 | Euth (acute phase) | 0.584667 | 0.29133 | **2.01** |

No polarization towards any response profile was observed with TcTPE antigenic stimulation and, as in group II, only in euthanasia (acute phase of ChD) a polarized response towards a Th1 or inflammatory profile was observed with a 2.0 ratio.

| IgG1/IgG2a ratio | | | | | | | |
|---|---|---|---|---|---|---|---|
| Time | TcTPE+mBCG (Group V) | | | Time | TcTPE+mBCG/Tc (Group VI) | | |
| | IgG1 | IgG2a | Ratio | | IgG1 | IgG2a | Ratio |
| Postvac/Preinf | 0.3554 | 0.234 | 1.52 | Postvac/Preinf | 0.362 | 0.2386 | 1.52 |
| Euthanasia time | 0.3662 | 0.2386 | 1.64 | Euth (acute phase) | 0.654 | 0.42675 | 1.53 |
| Mean IgG2a/IgG1 ratio | | | **1.58** | Mean IgG2a/IgG1 ratio | | | **1.52** |

In these groups (V and VI) the stimulation of the vaccine formulation caused a slight polarization (less than double) to a Th2 or anti-inflammatory response. Upon *T. cruzi* infection, this response persisted in an average of 1.5 ratio during all acute phase of ChD.

| IgG2a/IgG1 or IgG1/IgG2a ratios | | | | | | | |
|---|---|---|---|---|---|---|---|
| Time | TcTPE+wtBCG (Group III) | | | Time | TcTPE+wtBCG/Tc (Group IV) | | |
| | IgG1 | IgG2a | Ratio | | IgG1 | IgG2a | Ratio |
| Postvac/Preinf | 0.32175 | 0.2195 | **1.47** | Postvac/Preinf | 0.3278 | 0.2148 | **1.53** |
| Euthanasia time | 0.3145 | 0.221 | **1.42** | Euth (acute phase) | 0.48133 | 0.523 | 0.92 |

The stimulation with wtBCG adjuvanted vaccine formulation initially promoted a slight polarization of less than 1.5 times towards a Th1 or inflammatory response. When acute infection occurred, the host tried to restrain the inflammatory environment that was being generated via a mixed Th1/Th2 immune response of similar proportions (0.92 ratio); however, only 20% of the animals showed an improvement in their physical condition, while 80% died.

## Table S2. IFN-γ/IL-4 ratio

| IFN-γ / IL-4 ratio | | | |
|---|---|---|---|
| **Time** | **Tc (Group II)** | | |
| | IFN-γ | IL-4 | Ratio |
| Postvac/Preinf | 114.487 | 111.693 | 1.03 |
| Euth (acute phase) | 974.917 | 442.985 | **2.20** |

In group II, only in euthanasia (acute phase of ChD) a polarization to a Th1 or inflammatory response in a 2.2 ratio was observed, as *T. cruzi* infection has always been described at this stage of the disease.

| IFN-γ/IL-4 | | | | | | | |
|---|---|---|---|---|---|---|---|
| **Time** | **TcTPE (Group VII)** | | | **Time** | **TcTPE/Tc (Group VIII)** | | |
| | IFN-γ | IL-4 | Ratio | | IFN-γ | IL-4 | Ratio |
| Postvac/Preinf | 293.903 | 271.811 | 1.08 | Postvac/Preinf | 290.707 | 269.352 | 1.08 |
| Euthanasia time | 269.960 | 274.676 | 0.98 | Euth (acute phase) | 715.480 | 375.872 | **1.90** |

No polarization towards any response profile was observed with TcTPE antigenic stimulation and, as in group II, except in euthanasia (acute phase of ChD) when a polarized response towards a Th1 or inflammatory profile was observed, a 1.9 ratio was calculated, very similar to group II without immunization.

| IFN-γ/IL-4 ratios | | | | | | | |
|---|---|---|---|---|---|---|---|
| **Time** | **TcTPE+mBCG (Group V)** | | | **Time** | **TcTPE+mBCG/Tc (Group VI)** | | |
| | IFN-γ | IL-4 | Ratio | | IFN-γ | IL-4 | Ratio |
| Postvac/Preinf | 287.456 | 333.541 | 0.86 | Postvac/Preinf | 284.843 | 335.614 | 0.85 |
| Euthanasia time | 273.390 | 339.974 | 0.80 | Euth (acute phase) | 857.257 | 770.723 | 1.11 |

In these groups (V and VI) the stimulation of the vaccine formulation, did not cause predominance of any of these cytokines, since their production occurred in similar proportions (0.80-1.11 ratios). This vaccine formulation prevented the promotion of a pro-inflammatory environment caused by *T. cruzi* infection, which is consistent with what was observed in the production of IgG1>IgG2a levels.

| IFN-γ/IL-4 ratios | | | | | | | |
|---|---|---|---|---|---|---|---|
| **Time** | **TcTPE+wtBCG (Group III)** | | | **Time** | **TcTPE+wtBCG/Tc (Group IV)** | | |
| | IFN-γ | IL-4 | Ratio | | IFN-γ | IL-4 | Ratio |
| Postvac/Preinf | 295.93 | 312.936 | 0.95 | Postvac/Preinf | 292.480 | 313.640 | 0.93 |
| Euthanasia time | 275.483 | 314.362 | 0.88 | Euth (acute phase) | 854.54 | 573.081 | **1.49** |

No polarization towards any response profile was observed with wtBCG adjuvanted vaccine formulation, and when acute infection was present (in euthanasia), there was a slight polarization (1.49 ratio) towards Th1 type inflammatory response.

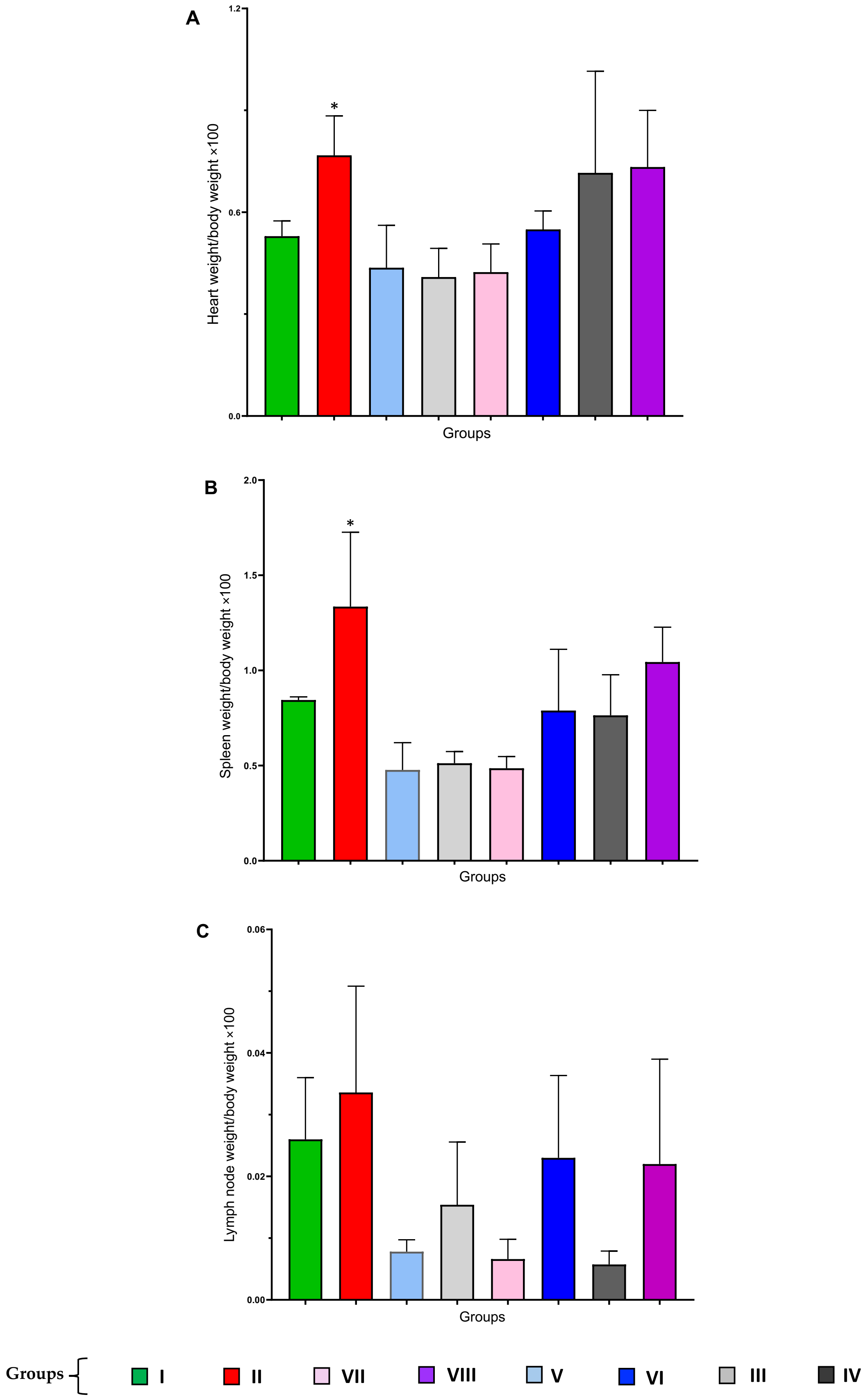
A
1.2
0.6
0.0
Heart weight/body weight ×100
*
Groups
B
2.0
1.5
1.0
0.5
0.0
Spleen weight/body weight ×100
*
Groups
C
0.06
0.04
0.02
0.00
Lymph node weight/body weight ×100
Groups
Groups
I
II
VII
VIII
V
VI
III
IV

Figure S2. Heart (a), splenic (b) and lymph node (c) indices with the vaccine formulation of TcTPE using *M. bovis* BCG strains as adjuvants and infected with *T. cruzi*. Values represent the mean ± SD of organic indices for group and are representative of two independent experiments with equivalent results. Kruskal-Wallis test was used for determining significant differences when (*) $p \leq 0.05$ when comparing all groups with the healthy group. Group I = not vaccinated/not infected; group II = not vaccinated/infected; group III = TcTPE-vaccinated+wtBCG as adjuvant/not infected; group IV = TcTPE-vaccinated+wtBCG as adjuvant/infected; group V = TcTPE-vaccinated+BCGΔBCG1419c as adjuvant/not infected; group VI = TcTPE-vaccinated+BCGΔBCG1419c as adjuvant/infected; group VII = TcTPE-vaccinated/not infected; group VIII = TcTPE-vaccinated/infected.
